# Supplementary material for: Effect of Antibacterial Prophylaxis on Febrile Neutropenic Episodes and Bacterial Bloodstream Infections in Dutch Pediatric Patients with Acute Myeloid Leukemia: A Two-Center Retrospective Study
Source: Cancers (Basel). 2022 Jun 28;14(13):3172. doi: 10.3390/cancers14133172 (PMC9264841; doi:10.3390/cancers14133172)
Supplement: Supplementary file 1 [file cancers-14-03172-s001.zip › cancers-1788751-supplementary.pdf]

Supplementary materials

Table S1. Protocol details of the four consecutive Dutch Childhood Oncology Group treatment protocols.

**Chemotherapy phases**	**ANLL-97/AML-12**	**AML-15**	**DB-AML-01**	**NOPHO-DBH AML 2012**
Induction course I	*ADE 10+3+5*<br>- Cytarabine 100 mg/m² every 12 hours, days 1-10 (cumulative dose 2000 mg/m²)<br>- Daunorubicin 50 mg/m² per day in 6-hour infusion, days 1, 3, 5<br>- Etoposide 100 mg/m² per day in 4-hour infusion, days 1-5<br><br>*MAE*<br>- Mitoxantrone 12 mg/m² per day, days 1, 3, 5<br>- Cytarabine 100 mg/m² every 12 hours, days 1-10 (cumulative dose 2000 mg/m²)<br>- Etoposide 100 mg/m² per day, days 1-5	*ADE 10+3+5*<br>- Cytarabine 100 mg/m² every 12 hours, days 1-10 (cumulative dose 2000 mg/m²)<br>- Daunorubicin 50 mg/m² per day in 1-hour infusion, days 1, 3, 5<br>- Etoposide 100 mg/m² per day in 4-hour infusion, days 1-5<br><br>*FLAG-Ida*<br>- Fludarabine 30 mg/m² per day in 30-minute infusion, days 2-6<br>- Cytarabine 2 g/m² per day in 4-hour infusion, days 2-6 (cumulative dose 10000 mg/m²)<br>- Idarubicin 8 mg/m² per day in 1-hour infusion, days 4-6<br>- G-CSF 5 μg/kg per day in 30-minute infusion, days 1-7	*AIET*<br>- 6-Thioguanine 100 mg/m² every 12 hours orally, days 1-4<br>- Cytarabine 200 mg/m2 per day continuous infusion, days 1-4 (cumulative dose 800 mg/m²)<br>- Etoposide 100 mg/m² per day continuous infusion, days 1-4<br>- Idarubicin 12 mg/m² per day in 4-hour infusion, days 2, 4, 6	*MEC*<br>- Mitoxantrone 5 mg/m² per day in 1-hour infusion, days 6-10<br>- Etoposide 150 mg/m² per day in 2-hour infusion, days 1-5<br>- Cytarabine 200 mg/m² per day in 12-hour infusion, days 6-12 (cumulative dose 1400 mg/m²)<br><br>*DxEC*<br>- Daunoxome® 60 mg/m² per day in 1-hour infusion, days 6, 8, 10<br>- Etoposide 150 mg/m² per day in 2-hour infusion, days 1-5<br>- Cytarabine 200 mg/m² per day in 12-hour infusion, days 6-12 (cumulative dose 1400 mg/m²)
Induction course II	*ADE 8+3+5*<br>- Cytarabine 100 mg/m² every 12 hours, days 1-8 (cumulative dose 1600 mg/m²)<br>- Daunorubicin 50 mg/m² per day in 6-hour infusion, days 1, 3, 5	*ADE 8+3+5*<br>- Cytarabine 100 mg/m² every 12 hours, days 1-8 (cumulative dose 1600 mg/m²)<br>- Daunorubicin 50 mg/m² per day in 1-hour infusion, days 1, 3, 5	*AM*<br>- Cytarabine 100 mg/m² per day continuous infusion, days 1-5 (cumulative dose 500 mg/m²)<br>- Mitoxantrone 10 mg/m² per day in 30-minute infusion,	*ADxE*<br>- Cytarabine 100 mg/m² continuous infusion, days 1-2 (cumulative dose 200 mg/m²)<br>- Cytarabine 100 mg/m² every 12 hours in 30-minute infusion, days 3-8 (cumulative dose 1200

	- Etoposide 100 mg/m² per day in 4-hour infusion, days 1-5<br><br>*MAE*<br>- Mitoxantrone 12 mg/m² per day, days 1, 3 ,5<br>- Cytarabine 100 mg/m² every 12 hours, days 1-8 (cumulative dose 1600 mg/m²)<br>- Etoposide 100 mg/m² per day, days 1-5	- Etoposide 100 mg/m² per day in 4-hour infusion, days 1-5<br><br>*FLAG-Ida*<br>- Fludarabine 30 mg/m² per day in 30-minute infusion, days 2-6<br>- Cytarabine 2 g/m² per day in 4-hour infusion, days 2-6 (cumulative dose 10000 mg/m²)<br>- Idarubicin 8 mg/m² per day in 1-hour infusion, days 4-6<br>- G-CSF 5 µg/kg per day in 30-minute infusion, days 1-7	days 1-3<br><br>*FLADx*<br>- Fludarabine 30 mg/m² per day in 30-minute infusion, days 1-5<br>- Cytarabine 2 g/m² per day in 4-hour infusion, days 1-5 (cumulative dose 10000 mg/m²)<br>- Daunoxome® 60 mg/m² per day in 4-hour infusion, days 2, 4, 6	mg/m²)<br>- Daunoxome® 60 mg/m² per day in 1-hour infusion, days 2, 4, 6<br>- Etoposide 150 mg/m² per day in 2-hour infusion, days 6-8<br><br>*FLADx*<br>- Fludarabine 30 mg/m² per day in 30-minutes infusion, days 1-5<br>- Cytarabine 2 g/m² per day in 3-hour infusion, days 1-5 (cumulative dose 10000 mg/m²)<br>- Daunoxome® 60 mg/m² per day in 1-hour infusion, days 2, 4, 6
Consolidation course I	*MACE*<br>- Amsacrine 100 mg/m² per day in 1-hour infusion, days 1-5<br>- Cytarabine 200 mg/m² per day in 19-hour infusion, days 1-5 (cumulative dose 1000 mg/m²)<br>- Etoposide 100 mg/m² per day in 4-hour infusion, days 1-5	*MACE*<br>- Amsacrine 100 mg/m² per day in 1-hour infusion, days 1-5<br>- Cytarabine 200 mg/m² per day in 19-hour infusion, days 1-5 (cumulative dose 1000 mg/m²)<br>- Etoposide 100 mg/m² per day in 4-hour infusion, days 1-5<br><br>*HD-Ara-C*<br>- Cytarabine 3 g/m² every 12 hours in 4-hour infusion, days 1, 3, 5 (cumulative dose 18000 mg/m²)<br><br>*Allogeneic stem cell transplantation*	*HA$_2$E*<br>- Cytarabine 2 g/m² every 12 hours in 2-hour infusion, days 1-3 (cumulative dose 12000 mg/m²)<br>- Etoposide 100 mg/m² per day in 1-hour infusion, days 2-5	*HAM*<br>- Cytarabine 1 g/m² every 12 hours in 2-hour infusion, days 1-3 (cumulative dose 6000 mg/m²)<br>- Mitoxantrone 10 mg/m² per day in 1-hour infusion, days 3-5
Consolidation course II	*CLASP*<br>- Cytarabine 3 g/m² every 12	*MidAC*<br>- Mitoxantrone 10 mg/m² per	*HA$_3$*<br>- Cytarabine 3 g/m² every 12	*HA$_3$E*<br>- Cytarabine 3 g/m² every 12

	hours in 3-hour infusion, days 1-2, 8-9 (cumulative dose 24000 mg/m^2)<br>- L-Asparaginase 6000 E/m^2 in 1-hour infusion, days 2, 9<br><br>*MidAC*<br>- Mitoxantrone 10 mg/m^2 per day in 6-hour infusion, days 1-5<br>- Cytarabine 1 g/m^2 every 12 hours in 2-hour infusion, days 1-3 (cumulative dose 6000 mg/m^2)<br><br>*Allogeneic stem cell transplantation*	day in 1-hour infusion, days 1-5<br>- Cytarabine 1 g/m^2 every 12 hours in 2-hour infusion, days 1-3 (cumulative dose 6000 mg/m^2)<br><br>*HD-Ara-C*<br>- Cytarabine 3 g/m^2 every 12 hours in 4-hour infusion, days 1, 3, 5 (cumulative dose 18000 mg/m^2)	hours in 2-hour infusion, days 1-3 (cumulative dose 18000 mg/m^2)	hours in 2-hour infusion, days 1-3 (cumulative dose 18000 mg/m^2)<br>- Etoposide 100 mg/m^2 per day in 1-hour infusion, days 1-5<br><br>*Allogeneic stem cell transplantation*
Consolidation course III	*MidAC*<br>- Mitoxantrone 10 mg/m^2 per day in 6-hour infusion, days 1-5<br>- Cytarabine 1 g/m^2 every 12 hours in 2-hour infusion, days 1-3 (cumulative dose 6000 mg/m^2)<br><br>*Allogeneic stem cell transplantation*		*HA$_2$E*<br>- Cytarabine 2 g/m^2 every 12 hours in 2-hour infusion, days 1-3 (cumulative dose 12000 mg/m^2)<br>- Etoposide 100 mg/m^2 per day in 1-hour infusion, days 2-5	*FLA*<br>- Fludarabine 30 mg/m^2 per day in 30-minutes infusion, days 1-5<br>- Cytarabine 2 g/m^2 per day in 3-hour infusion, days 1-5 (cumulative dose 10000 mg/m^2)

Table S2. The incidence of febrile neutropenic episodes, bacterial bloodstream infections, and bacterial bloodstream infection-related pediatric intensive care unit admissions according to antibacterial prophylaxis regimen in pediatric acute myeloid leukemia patients excluding courses during which the antibacterial prophylaxis regimen switched during the neutropenic period.

**Antibacterial prophylaxis regimen**	**N of courses**	**FN episode**		***P***	**Bacterial BSI**		***P***	**Gram-positive BSI**		***P***	**VGS BSI**		***P***	**Gram-negative BSI**		***P***	**PICU**		***P***
		**N**	**%**		**N**	**%**		**N**	**%**		**N**	**%**		**N**	**%**		**N**	**%**	
Ciprofloxacin	85	77	90.6	**<0.0001**[†]	56	65.9	**<0.0001**[†]	51	60	**<0.0001**[†]	20	23.5	**0.001**[†]	7	8.2	**0.003**[†]	7	8.2	**0.029**[†]
Penicillin plus ciprofloxacin	127	98	77.2		43	33.9		42	33.1		17	13.4		1	0.8		3	2.4	
Teicoplanin plus ciprofloxacin	48	21	43.8		2	4.2		2	4.2		0	0		0	0		0	0	
Ciprofloxacin	85	77	90.6	**0.016**[‡]	56	65.9	**<0.0001**[‡]	51	60	**0.0001**[‡]	20	23.5	0.066[‡]	7	8.2	**0.008**[‡]	7	8.2	0.093[‡]
Penicillin plus ciprofloxacin	127	98	77.2		43	33.9		42	33.1		17	13.4		1	0.8		3	2.4	
Penicillin plus ciprofloxacin	127	98	77.2	**<0.0001**[‡]	43	33.9	**<0.0001**[‡]	42	33.1	**<0.0001**[‡]	17	13.4	**0.004**[‡]	1	0.8	1.000[‡]	3	2.4	0.563[‡]
Teicoplanin plus ciprofloxacin	48	21	43.8		2	4.2		2	4.2		0	0		0	0		0	0	
No prophylaxis	9	8	88.9	NA	5	55.6	NA	3	33.3	NA	2	22.2	NA	3	33.3	NA	1	11.1	NA
Penicillin	11	11	100	NA	6	54.5	NA	2	18.2	NA	2	18.2	NA	4	36.4	NA	3	27.3	NA
Macrolide plus ciprofloxacin	10	10	100	NA	7	70	NA	7	70	NA	2	20	NA	0	0	NA	1	10	NA

[†] Pearson's χ^2 test.

[‡] Fisher's exact test.

NOTE. Bold states statistical significance. Due to polymicrobial BSIs, the number of bacterial BSIs may not correspond with the accumulated number of Gram-positive and Gram-negative BSIs. Because of the small number of courses with no prophylaxis (*n*=9), single-agent penicillin (*n*=11), and a macrolide plus ciprofloxacin (*n*=10), these regimens were not statistically compared.

Abbreviations: BSI, bloodstream infection; FN, febrile neutropenic; N, number; NA, not applicable; PICU, pediatric intensive care unit; VGS, viridans group streptococci.

Table S3. The incidence of febrile neutropenic episodes, bacterial bloodstream infections, and bacterial bloodstream infection-related pediatric intensive care unit admissions according to antibacterial prophylaxis regimen in pediatric acute myeloid leukemia patients excluding courses during which empiric systemic antibiotics were initiated along with chemotherapy, but discontinued prior to the onset of prophylactic antibiotics.

**Antibacterial prophylaxis regimen**	**N of courses**	**FN episode**		***P***	**Bacterial BSI**		***P***	**Gram-positive BSI**		***P***	**VGS BSI**		***P***	**Gram-negative BSI**		***P***	**PICU**		***P***
		**N**	**%**		**N**	**%**		**N**	**%**		**N**	**%**		**N**	**%**		**N**	**%**	
Ciprofloxacin	51	45	88.2	**<0.0001†**	35	68.6	**<0.0001†**	31	60.8	**<0.0001†**	15	29.4	**0.002†**	4	7.8	**0.031†**	4	7.8	0.087†
Penicillin plus ciprofloxacin	96	67	69.8		30	31.3		29	30.2		15	15.6		1	1.0		2	2.1	
Teicoplanin plus ciprofloxacin	34	13	38.2		1	2.9		1	2.9		0	0		0	0		0	0	
Ciprofloxacin	51	45	88.2	**0.014‡**	35	68.6	**<0.0001‡**	31	60.8	**0.0004‡**	15	29.4	0.056‡	4	7.8	**0.049‡**	7	7.8	0.183‡
Penicillin plus ciprofloxacin	96	67	69.8		30	31.3		29	30.2		15	15.6		1	1.0		3	2.1	
Penicillin plus ciprofloxacin	96	67	69.8	**0.002‡**	30	31.3	**0.0004‡**	29	30.2	**0.001‡**	15	15.6	**0.011‡**	1	1.0	1.000‡	3	2.1	0.563‡
Teicoplanin plus ciprofloxacin	34	13	38.2		1	2.9		1	2.9		0	0		0	0		0	0	
No prophylaxis	8	7	87.5	NA	5	62.5	NA	3	37.5	NA	2	25	NA	3	37.5	NA	1	12.5	NA
Penicillin	9	9	100	NA	5	55.6	NA	2	22.2	NA	2	22.2	NA	3	33.3	NA	3	22.2	NA
Macrolide plus ciprofloxacin	12	9	75	NA	6	50	NA	6	50	NA	2	16.7	NA	0	0	NA	1	8.3	NA

† Pearson's χ^2 test.

‡ Fisher's exact test.

NOTE. Bold states statistical significance. Due to polymicrobial BSIs, the number of bacterial BSIs may not correspond with the accumulated number of Gram-positive and Gram-negative BSIs. Because of the small number of courses with no prophylaxis (*n*=8), single-agent penicillin (*n*=9), and a macrolide plus ciprofloxacin (*n*=12), these regimens were not statistically compared.

Abbreviations: BSI, bloodstream infection; FN, febrile neutropenic; N, number; NA, not applicable; PICU, pediatric intensive care unit; VGS, viridans group streptococci.
